# Functional Outcomes of Posterior Plate Fixation in Complex Tibial Plateau Fractures: A Retrospective Study

**DOI:** 10.7759/cureus.83167

**Published:** 2025-04-29

**Authors:** Nikhilesh Das, Suman S Mishra, Anuraag Mohanty, Dhananjay Sahoo

**Affiliations:** 1 Department of Orthopaedics, Peerless Hospital and B.K. Roy Research Centre, Kolkata, IND; 2 Department of Orthopaedics, Kalinga Institute of Medical Sciences, Bhubaneswar, IND; 3 Department of Orthopaedics, Apollo Hospitals, Bhubaneswar, Bhubaneswar, IND

**Keywords:** functional outcomes, knee society score (kss), posterior plate fixation, posterior tibial plateau fracture (ptpf), radiological outcomes

## Abstract

Aim

The research aimed to assess the functional and radiological results of posterior plate fixation in complex types of posterior tibial plateau fractures (PTPFs) with special regard to the stability of the region, healing of the fractures, and the patient's functioning several years after surgery.

Materials and methods

An exhaustive study of the concerned group of patients was done on 30 individuals who underwent posterior plate fixation for closed PTPFs at the Department of Orthopaedics and Traumatology, Peerless Hospital and B.K. Roy Research Centre, Kolkata, India, from September 2018 to March 2021. A thorough clinical and radiological follow-up was carried out for every patient at set periods of time, before and after the operation. After a thorough study, key outcome metrics were measured, such as duration of fracture union, articular congruity, Knee Society Scores (KSS), International Knee Documentation Committee (IKDC) scores, range of motion (ROM) of the knee, and complications.

Results

The study included 30 participants, with a mean age of 40.70 ± 8.46 years. These included 25 males and 5 females. All fractures showed radiological union after a mean of 16.2 ± 3.1 weeks. Excellent articular congruity (≤2 mm step-off) was achieved in 29 (96.6%) cases. The mean clinical and functional KSS was 85.60 ± 7.09 and 81.23 ± 6.09, respectively; the mean IKDC score was 70.83 ± 6.92. Mean knee flexion achieved was 122.60° ± 8.08°, and all patients achieved independent ambulation at a mean of 18.1 ± 2.6 weeks. Complications managed conservatively included superficial infection in three (10%) patients and knee stiffness in four (13.3%) patients.

Conclusion

Posterior plate fixation has proven to be simple and effective, achieving excellent radiological and functional outcomes. Patients were able to gain early movement and independent walking by the final follow-up, with all patients having stable fixation. The technique was reliable, with low complication rates and no implant failures or nonunion, and was an effective surgical treatment for complex PTPFs.

## Introduction

Fractures of the tibial plateau account for roughly 1% of all fractures and are among the most intricate, as they can affect the knee’s stability, load-bearing, and function. For every 100,000 individuals, approximately 10.3 fractures occur each year, with the average age of patients being 52.6 years [1]. These fractures have a bimodal distribution: men younger than 50 years usually sustain them due to high-energy trauma, while women older than 70 years are more prone to fractures from low-energy falls resulting from osteoporotic bone changes [2,3]. Among the many types of tibial plateau fractures, posterior tibial plateau fractures (PTPFs) are among the most difficult, occurring in nearly 28.8% of bicondylar fractures. If PTPFs are not treated appropriately, they can lead to posterior tibial subluxation, progressive joint incongruity, and joint instability [4].

Anterolateral plating is one of the popular approaches used in traditional fixation methods; however, it does not offer proper containment for posterior column fractures, which results in high rates of articular collapse and restricted recovery. Luo's approach, known as the Three Column Concept, provides a better framework for understanding tibial plateau fractures by suggesting the need for specific fixation designs suited to the fracture form [5]. Biomechanical studies have shown that posterior plating offers better resistance to shear forces and prevents the subsidence of posterior fragments, which is often a problem in both anterolateral and two-stage approaches. Clinical outcomes have shown that patients treated with posterior plating experience better functional results, with joint congruity and earlier mobility. Nonetheless, these approaches require greater surgical skill, longer operative times, and carry the risk of soft tissue injury, warranting close attention to their long-term outcomes [6].

This study aims to assess the functional and radiological outcomes of posterior plate fixation in PTPFs, hypothesizing that direct stabilization of the posterior column will achieve improved knee stability, increased potential for rehabilitation, and fewer long-term complications, compared to traditional fixation techniques.

## Materials and methods

Study design and setting

This retrospective study was conducted at the Department of Orthopaedics and Traumatology, Peerless Hospital and B.K. Roy Research Centre, in Kolkata, India. The patients were seen between September 2018 and March 2021. We included adults aged 18-70 who had closed PTPFs confirmed by CT scans (with 3D views). Patients were excluded if they had open fractures, multiple severe injuries, significant pre-existing knee problems, or neuromuscular disorders.

Every patient underwent posterior plating surgery using a consistent technique. We followed up with them for at least a year after surgery. Checkups, including both physical exams and X-rays, were conducted at 2, 6, 12, and 24 weeks, and then again at 6 and 12 months. For the X-rays, we assessed how well the fracture had healed and how well the joint surfaces were aligned. To evaluate functional outcomes, we used Knee Society Scores (KSS), International Knee Documentation Committee (IKDC) scores, and measured the knee’s range of motion (ROM) [7]. Because we carefully assessed outcomes and used a standardized surgical approach, we were able to reliably evaluate the effectiveness and safety of posterior plating for these difficult fractures.

Inclusion criteria

We accepted patients aged 18 to 70 years. They were required to have a closed posterior column tibial plateau fracture, and we verified this with CT scans with 3D reconstructions. Patients who had definitive surgical treatment by posterior plate fixation were included. Patients with an intact, functioning contralateral limb for proper evaluation of the functional results were included. Patients must be reasonable and able to sustain a follow-up period of at least 12 months, involving assessments both clinically and radiologically.

Exclusion criteria

To enhance clarity and minimize confounding factors, patients exhibiting open tibial plateau fractures, polytrauma, and soft tissue damage needing external fixation or staged treatment were excluded. Patients with certain neuromuscular disorders that impact the lower limb were also excluded, as these disorders would impact functional outcomes independently. Patients with advanced pre-existing knee problems, particularly osteoarthritis or other forms of significant joint pathology, were excluded in order to mitigate any potential confounding impacts on the clinical outcome evaluation. Subjects lacking the willingness or ability to attend multiple postoperative evaluations 12 months after surgery were excluded in order to guarantee outcome evaluation reliability.

Preoperative assessment

**Figure 1:**
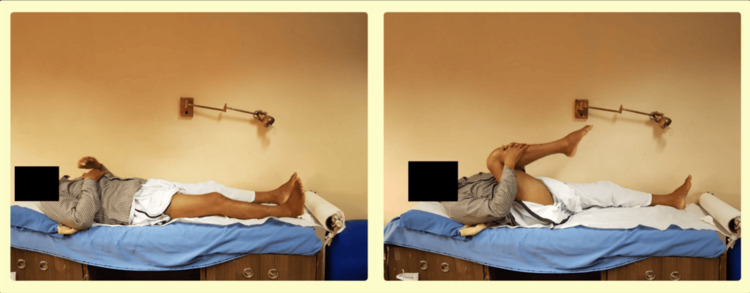
Clinical demonstration of hip and knee flexion assessment in the supine position. These images reflect the general technique used to assess lower limb joint mobility. In patients with proximal tibial fractures, assessments were tailored based on pain tolerance and fracture stability, with appropriate precautions to avoid stress at the injury site.

**Figure 2:**
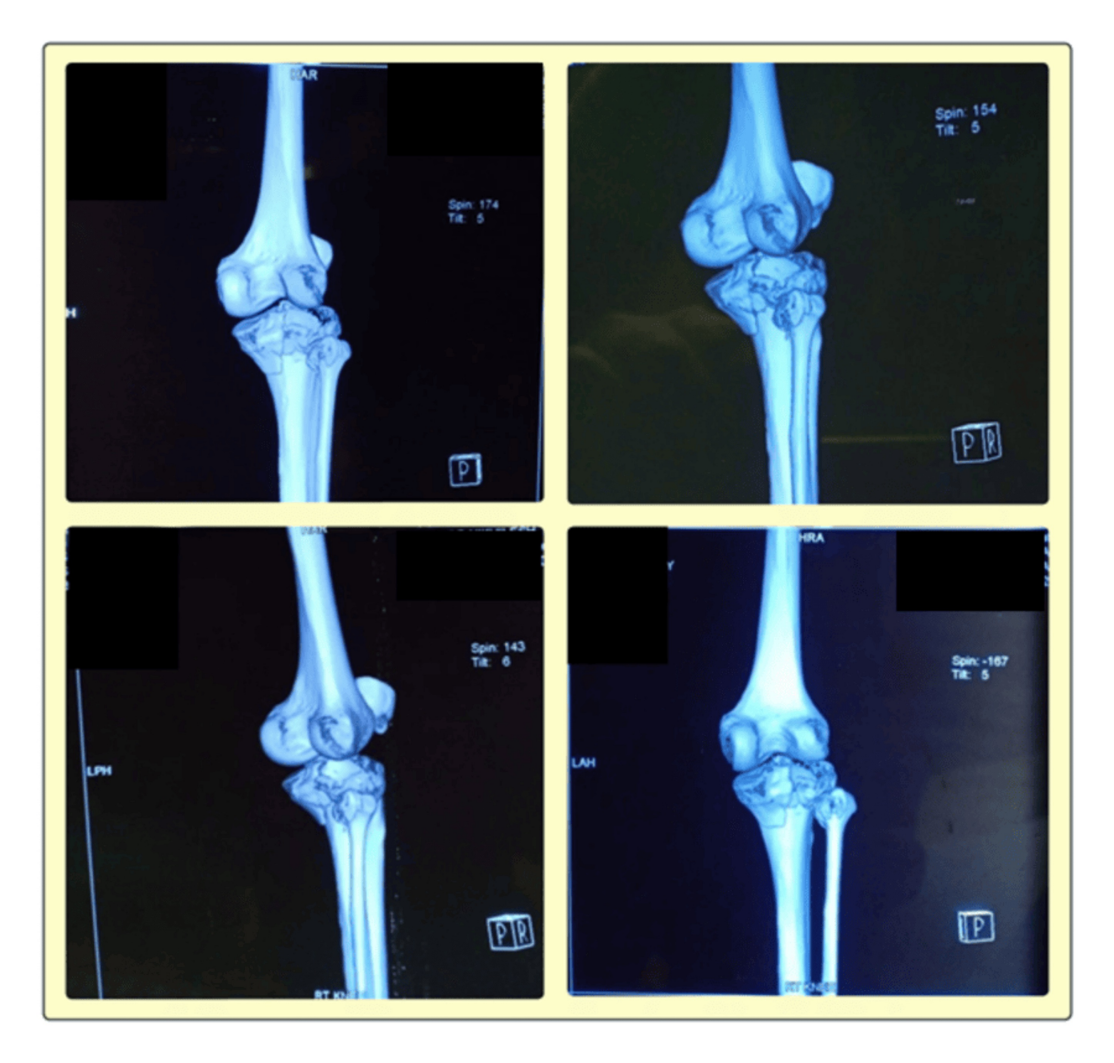
3D CT scan images reveal a comminuted intra-articular fracture of the proximal tibia with significant displacement. These scans provide critical insights into the fracture pattern, joint involvement, and surgical planning, for optimal fixation and functional recovery.

Patients were evaluated comprehensively, taking into account their injury history, mechanism of trauma, and soft tissue injury, for clinical examination to estimate severity, check for possible complications, and inform the surgical plan (Figure 1). AP and lateral knee X-rays were done as part of the initial assessment of the fractures, which provided essential preliminary data for the diagnosis of the fractures. Preoperative planning, along with the selection of the implant and the surgical approach, was done with meticulous attention to detail, facilitated by routine CT scans with 3D reconstruction in order to accurately define the fracture pattern, posterior column involvement, and any other accompanying articular depression of the fracture (Figure 2). The patient’s overall health status and fitness for surgery, aimed at reducing complications, were established through routine preoperative tests such as CBC, coagulation profile, kidney function tests, and ECG. Patients were provided with comprehensive information regarding their surgical procedures, including expected outcomes, rehabilitation protocols, and complications, with the aim of improving patient compliance, rehabilitation, and expectations to accomplish favorable surgical outcomes.

Surgical approaches

In this study, we selected the surgical approach for fixation according to the fracture type, soft tissue condition, and the need to visualize the fracture pieces for accurate anatomical reduction.

Posteromedial Approach

This approach is primarily used for fractures of the posterior medial column. It requires dissection through the space between the pes anserinus and the medial head of the gastrocnemius muscle. Using this technique, a stable and accurate reduction and fixation can be achieved with minimal internal and external soft tissue exposure.

Posterior Inverted L-shaped Approach

This technique was selected for complicated bicondylar fractures with significant posterior fragment displacement. It enabled viewing of both the posteromedial and lateral tibial plateau fragments simultaneously and facilitated strong, anatomically accurate reduction and fixation [8].

Modified Posterolateral Approach Without Fibular Osteotomy

This approach was preferred in cases of fractures primarily involving the posterolateral column. The lateral fracture fragments were approached directly through the space between the biceps femoris and the lateral head of the gastrocnemius muscle. These procedures were performed without fibular osteotomy, allowing preservation of the lateral knee stability and reducing surgical impact [9].

Surgical procedure

The surgical technique varied slightly depending on the chosen approach, but the core principles of exposure, reduction, and fixation remained consistent. All procedures were performed under general or regional anesthesia, with the use of a tourniquet in selected cases.

For the posteromedial approach, the patient was positioned supine, with the leg externally rotated and supported to allow access to the posteromedial aspect of the tibia. A straight or gently curved longitudinal incision was made along the posteromedial border of the proximal tibia. The subcutaneous tissue was dissected, and the pes anserinus was retracted anteriorly. The medial head of the gastrocnemius was retracted posteriorly to expose the posteromedial cortex. The posterior capsule was opened, if required, to visualize the articular surface, and any hematoma or debris was cleared. Fracture fragments were reduced under direct vision, and any metaphyseal defects were grafted as needed.

For the posterior inverted L-shaped approach, the patient was placed in a prone position, with a bump under the ankles to slightly flex the knee, relaxing the posterior soft tissues. A transverse incision was made along the popliteal crease and extended distally and medially along the posteromedial aspect of the proximal tibia to form an inverted “L” shape. Subcutaneous tissue was dissected carefully to preserve cutaneous perforators. The deep fascia was incised along the same line, and the heads of the gastrocnemius muscle were mobilized using blunt dissection. The neurovascular bundle, situated centrally or slightly posterolaterally in the popliteal fossa, was identified and protected throughout the procedure. The posterior joint capsule was incised either vertically or obliquely near the fracture site, and the joint was irrigated to remove hematoma. Depressed articular fragments were reduced using a small elevator or bone tamp, and alignment was confirmed both directly and fluoroscopically. Voids were filled with either autograft or allograft, as required.

In the modified posterolateral approach, the patient was placed in the prone or lateral decubitus position, depending on the surgeon's preference and equipment availability. A posterolateral incision was made, starting proximal and posterior to the fibular head and extending distally along the posterolateral aspect of the tibia. Dissection was carried out through the interval between the biceps femoris and the lateral head of the gastrocnemius muscle. The common peroneal nerve was identified proximally and carefully protected throughout the procedure. The lateral head of the gastrocnemius was gently retracted medially to expose the posterolateral tibial cortex. Capsulotomy was performed when necessary to access intra-articular fragments. Reduction was performed under direct vision, and metaphyseal voids were addressed using a bone graft.

In all approaches, provisional fixation was achieved using Kirschner wires (K-wires) or reduction clamps. The reduction was confirmed under fluoroscopy in multiple planes. Once satisfactory alignment was ensured, definitive fixation was performed using pre-contoured posterior buttress plates or low-profile locking plates adapted to the posterior surface of the tibia. Plates were secured using subchondral locking screws and cortical screws, placed under fluoroscopic control to avoid intra-articular penetration.

Following fixation, the surgical field was irrigated thoroughly. The joint capsule, where incised, was closed with absorbable sutures. The deep fascia was approximated over the gastrocnemius muscle, and layered closure of the subcutaneous tissue and skin was performed using absorbable and non-absorbable sutures, respectively. In cases with extensive soft tissue dissection or anticipated fluid accumulation, a closed-suction drain was placed and removed within 24-48 hours postoperatively.

Fixation strategy

The fixation strategy used in the above study focused on achieving optimal anatomical restoration, along with stable fixation and joint congruity. These are very important in the functional recovery of complex PTPFs [10]. To counter muscle spasm and enable fragmented parts realignment, manual manipulation was first performed with traction. Precise muscle realignment was done to minimize joint incongruity and lower the chance of osteoarthritis later on. Next, the fracture fragments were stabilized with manual K-wires after manual reduction. Definitive fixation was done with posterior buttress plates molded to the posterior tibial surface, which were the most effective, as they resisted posterior shear forces. Through the critical healing phase, these plates ensured the integrity of the reduction, maintaining it during healing. Stable fixation was provided, which helps early functional recovery, by securing the plates with locking and cortical screws [11].

Postoperative protocol

**Figure 3:**
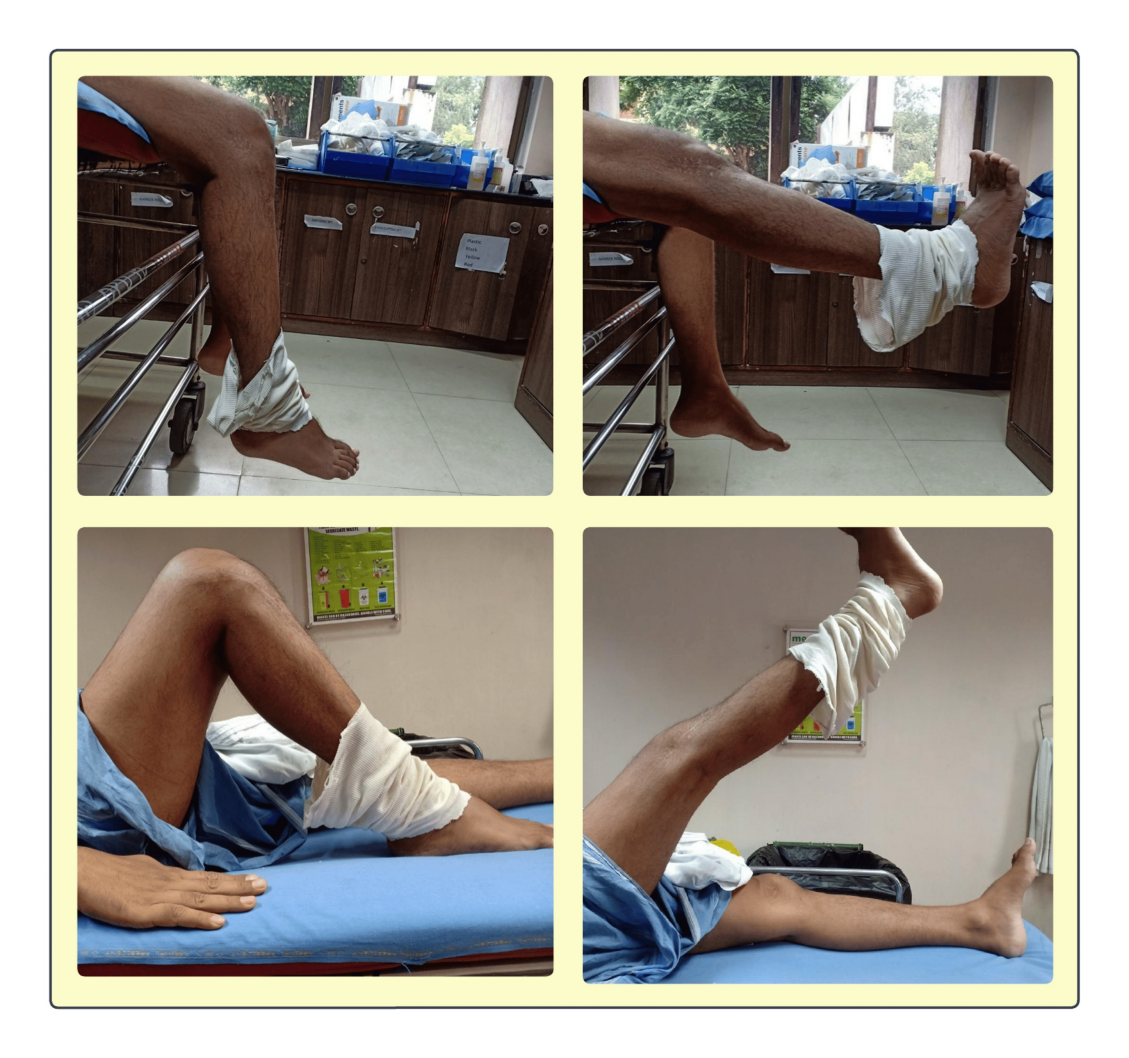
The patient performs a series of controlled movements, including knee flexion, extension, and straight leg raises, to restore mobility, strengthen muscles, and improve functional outcomes following surgical intervention. Top Left: Early-stage passive dangling of the limb from the bed to initiate gravity-assisted knee flexion and improve circulation; Top Right: Active-assisted elevation demonstrating controlled muscle activation and partial range of motion to facilitate quadriceps engagement; Bottom Left: Supine knee bending, a key exercise to regain knee flexion, maintain joint mobility, and prevent stiffness; Bottom Right: Straight leg raises to strengthen the quadriceps and hip flexors, crucial for independent ambulation and joint stabilization.

Radiographs were performed immediately post-surgery to check for any misalignment of the fracture and the position of the implants that may need adjustments. Soft tissues were protected by extending the knee with a brace for two weeks and immobilizing the knee. To maintain joint range and mobility and avoid stiffness, passive knee ROM exercises were started two weeks after surgery. Crutch-assisted mobility with partial weight-bearing started at six weeks and was guided to full weight-bearing at 16 to 20 weeks, based on visual assessments of fracture healing, which was supported by the X-rays. For physiotherapy, a supervised rehabilitation program was designed with a focus on the quadriceps, proprioception, and gait training, combined with optimal functional activities to make the restoration of normal function more efficient (Figure 3).

Statistical analysis

Continuous variables, such as time to union and functional scores, were analyzed using Student’s t-test. Categorical variables, such as infection rates and complications, were analyzed using the Chi-square test or Fisher’s exact test, as appropriate. Statistical significance was set at p < 0.05.

Ethical considerations

This study was approved by the Institutional Review Board (IRB) of Peerless Hospital and B.K. Roy Research Centre (approval no. PHH&RCL-CREC/S09/2021). All patients provided written informed consent before participation. No external funding was received, and there were no conflicts of interest. This comprehensive methodology ensures standardized patient selection, consistent surgical techniques, and objective outcome assessments, allowing for an accurate evaluation of the role of posterior plating in tibial plateau fractures.

## Results

Demographic and clinical data

This study included a total of 30 patients with PTPFs that were managed with posterior plate fixation. The average age was 40.70 ± 8.46 years, and there was a marked male predominance, with 25 males and 5 females in the sample. The majority of injuries, 28 patients (96.67%), were attributed to road traffic accidents, while falls from height made up the other two patients (3.3%) (Table 1).

**Table 1:** This table summarizes the demographic characteristics, surgical parameters, and post-operative functional outcomes of patients who underwent posterior plate fixation for complex tibial plateau fractures. Statistical analysis was performed using one-sample t-tests for continuous variables and Chi-square or binomial tests for categorical variables. A p-value of <0.05 was considered statistically significant.

Parameter	Value	p-value
Total Patients	30	-
Mean Age (Years) (Mean ± SD)	40.70 ± 8.46	>0.05 (1)
Male-to-Female Ratio	25:5	<0.05 (0.0003)
Mechanism of Injury	93.3% Road Traffic Accidents (RTAs), 6.6% Falls	<0.05 (0)
Mean Operative Time (Minutes) (Mean ± SD)	123.97 ± 11.56	>0.05 (0.9988)
Mean Time to Union (Weeks) (Mean ± SD)	16.10 ± 2.01	>0.05 (1)
Residual Articular Step-Off <=2mm (%)	96.67%	<0.05 (0)
Knee Society Score (KSS) - Clinical (Mean ± SD)	85.60 ± 7.09	>0.05 (1)
Knee Society Score (KSS) - Functional (Mean ± SD)	81.23 ± 6.09	>0.05 (0.9976)
International Knee Documentation Committee (IKDC) Score (Mean ± SD)	70.83 ± 6.92	>0.05 (0.9979)
Mean Knee Flexion (Degrees) (Mean ± SD)	122.60 ± 8.08	>0.05 (1)
Wound Infection (%)	10	<0.05 (0)
Knee Stiffness (%)	13.3	<0.05 (0)
Implant Failure (%)	0	<0.05 (0)
Nonunion (%)	0	<0.05 (0)

Operative parameters

The average operative time was 123.97 ± 11.56 minutes. The mean days from injury to surgical intervention was 6.2 ± 2.1 days, and the average intraoperative fluoroscopy time was 3.8 ± 1.2 minutes. All patients had satisfactory fracture reduction and alignment during the operation, which were confirmed by postoperative radiographs. The distribution of complications varied slightly across age groups, with a slightly higher rate of knee stiffness noted in patients aged 40-50 years (Table 2).

**Table 2:** This table outlines the frequency of postoperative complications, superficial infection, knee stiffness, implant failure, and nonunion, stratified by patient age groups. It helps assess age-related trends in complication incidence following posterior plate fixation.

Age Group	No. of Patients	Superficial Infection	Knee Stiffness	Non-union	Implant Failure	p-value (Infection & Stiffness)
<40 years	12	1	1	0	0	>0.05 (0.91)
40-50 years	10	1	2	0	0	
>50 years	8	1	1	0	0	

Radiological outcomes

**Figure 4:**
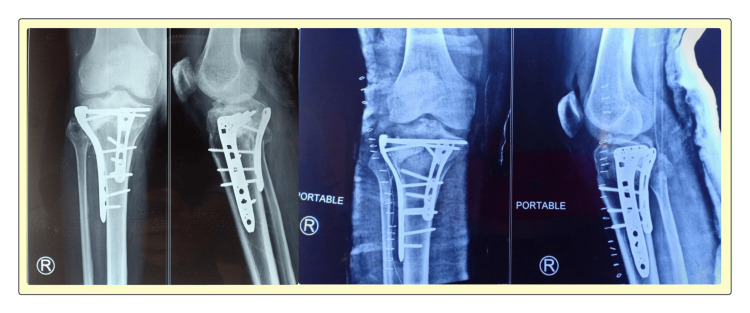
X-ray images show successful internal fixation using locking compression plates and screws, ensuring fracture stabilization and alignment. These images help assess implant positioning, bone healing progression, and potential complications during the recovery period.

As reassured by radiological examination, fracture union was accomplished at a mean time of 16.10 ± 2.01 weeks and was confirmed by cortical bridging that involved at least three of the four bone cortices. The efficiency of the union highlights the stability of the fixation and the process of healing. Moreover, residual articular step-off was ≤2 mm in 96.6% of patients who were scanned by CT at the final follow-up. This considerable reduction is indicative of the minimal step-off, which is essential in averting joint incongruity, early arthritis, and functional deficits. Radiological examination confirmed fracture union and proper alignment of internal fixation implants, as evidenced by postoperative X-ray imaging (Figure 4).

Functional outcomes

The average Clinical KSS associated with pain was 85.60 ± 7.09, depicting that most patients have small amounts of pain, good knee stability, and satisfactory ROM. A score in the 80-100 range demonstrates excellent knee function with no pain, as well as adequate stability. The mean Functional KSS grade was 81.23 ± 6.09, which suggests high levels of independence among patients in daily activities, including walking, climbing stairs, and general ambulation. On the other hand, the mean score of patients’ self-reported outcome was 70.83 ± 6.92 for the IKDC, which suggests a satisfying knee with few restrictions. Another key functional index, the ROM, was on average 122.60° ± 8.08° for knee flexion. Achieving over 120° flexion highlights a highly functional knee suitable for most everyday tasks and recreational activities. Functional scores and complication rates varied slightly depending on the surgical approach used, with the posteromedial approach showing the highest functional outcomes (Table 3).

**Table 3:** This table presents a comparative summary of the three primary surgical approaches utilized for posterior tibial plateau fracture fixation: the Posteromedial Approach, the Posterior Inverted L-Shaped Approach, and the Modified Posterolateral Approach, without fibular osteotomy. KSS, Knee Society Score; ROM, Range of Motion

Approach Used	No. of Cases	Mean KSS	Mean ROM	Complications	p-value (KSS)	p-value (ROM)
Posteromedial	12	86.1	124.2°	1 (8.3%)	>0.05 (0.16)	>0.05 (0.103)
Inverted L	10	83.5	121.0°	2 (20%)		
Modified PL	8	84.7	122.8°	1 (12.5%)		

Complications

Wound infections occurred in three (10%) of the patients. However, these were superficial and of no significant concern long-term, resolving with conservative management. Stiffness of the knee was noted in four (13.3%) of the patients at three months postoperatively, where patients had knee flexion of less than 90°. In most cases, physiotherapy resolved the stiffness, demonstrating the significance of early rehabilitation. Interestingly, there were no cases of implant failure or nonunion, illustrating the long-term effectiveness of the surgical fixation, as well as the healing of the fracture.

Summary of key findings

Patients who received posterior plating demonstrated an impressive functional recovery. They mobilized early and healed exceptionally well postoperatively. The mean time to fracture union for patients treated with posterior plating was 16.10 ± 2.01 weeks. Nearly all patients (93.3%) had minimal residual articular incongruity, with a step-off of 2 mm or less. Functional recovery was satisfactory, with the Clinical and Functional KSS at 85.60 ± 7.09 and 81.23 ± 6.09, respectively, and an average IKDC score of 70.83 ± 6.92. Postoperatively, around 18 weeks, patients attained independent ambulation and full weight-bearing, recovering an average knee flexion of 122.60° ± 8.08°. Superficial infection and transient knee stiffness were the most common complications, occurring in three (10%) patients and four (13.3%) patients, respectively. However, there were no cases of deep infection, implant failure, nonunion, or abnormal knee motion tracking.

Posterior plate fixation is a foremost candidate for surgical treatment of complex PTPFs, facilitating early rehabilitation, and, in turn, favorable long-term outcomes.

## Discussion

In this retrospective study, we describe the strikingly positive outcomes of posterior plate fixation of complex PTPFs in terms of bony fixation stability, anatomical restoration of the joint surface, and early mobilization with good short-term results. The mean time to fracture union following posterior plating was strikingly rapid (16.10 ± 2.01 weeks), and an articular step-off of ≤2 mm was maintained in 29 (96.67%) of patients, demonstrating a remarkable reduction in incongruity. Functional scores measured with the KSS (clinical: 85.60 ± 7.09 and functional: 81.23 ± 6.09) and IKDC (70.83 ± 6.92) showed markedly good knee function and quality of life, with average knee flexion at 122.60° ± 8.08°, and achievement of full independent ambulation consistently. Supported by the Three-Column Concept proposed by Wang et al., this approach aligns with the emerging literature about focusing fixation on posterior column fractures, with dual-plate techniques proposed by Patel et al., and rigorous surgical planning by Kottmeier et al. [5,12,13]. Radiologically, findings of excellent articular congruency, muscle strength, and endurance, according to meta-analysis, confirm that posterior plating provides the benefits of direct intraoperative visualization, accurate fragment reduction, and prevention of post-traumatic osteoarthritis in the posterior compartment. Use of posterior buttress plating provides adequate mechanical support by resisting shear stresses and avoiding the subsidence of posterior fragments, as stated by Zeng et al. [14], and clinically observed here via the absence of secondary displacement or implant failure. While infection at the surgical site was seen in three (10%) of cases (all of whom improved without surgery), and knee stiffness was noted in four (13.3%) of patients (who mostly improved with physiotherapy), these complications do not undermine the dominant benefits of early mobilization and stable fixation. Regardless, the findings cannot be generalized due to the small sample size, retrospective nature of the study, absence of a control group, and limited follow-up duration. Heretofore, longer-term and economic evaluations of the impact of posterior plate fixation are required, starting with prospective randomized studies for complete understanding. These outcomes suggest that posterior plating can provide solid fixation, optimal clinical and radiological outcomes, and swift recuperation from surgery in patients with PTPFs.

Considering the evidence provided, posterior plate fixation represents a very useful surgical treatment for PTPFs, as it provides good internal fixation, accurate anatomical reduction, and allows early mobilization. Its clinical efficacy, especially in complex fracture patterns, is further supported by the extensive literature reporting excellent radiological and functional outcomes, alongside low complication and implant failure rates. Even though quite optimistic, these results still require larger, prospective trials with longer follow-up periods to confirm long-term advantages, monitor post-traumatic arthritis risk, and evaluate cost-effectiveness. Nevertheless, as of now, posterior plating is perhaps the most reliable and practical treatment option for these patients, marking a significant advancement in the treatment of complex tibial plateau fractures [15].

This study has several limitations worth acknowledging. Firstly, the retrospective nature and relatively small sample size (n = 30) could limit the generalizability and statistical robustness of the results. Secondly, the absence of a control group, comparing posterior plating with alternative fixation techniques, prevents direct comparative analysis. Additionally, the short-term follow-up period (up to 12 months) does not allow a comprehensive assessment of long-term outcomes, such as late-onset osteoarthritis or functional deterioration. Further prospective randomized studies with larger sample sizes and extended follow-up periods are needed to validate these findings comprehensively.

## Conclusions

Posterior plate fixation provides reliable and stable fixation for complex PTPFs, consistently achieving excellent radiological and functional outcomes. Patients benefited from rapid fracture healing, precise anatomical reduction, and early mobilization, ultimately regaining independent ambulation. The low complication rate, absence of implant failures, and successful management of minor complications further highlight its safety and efficacy. This surgical approach significantly enhances patient outcomes, supporting its widespread adoption as a valuable treatment method for complex PTPFs.
